# Chronic comorbidities in persons living with HIV within three years of exposure to antiretroviral therapy at Pantang Antiretroviral Center in Ghana: a retrospective study

**DOI:** 10.11604/pamj.2022.42.294.35134

**Published:** 2022-08-19

**Authors:** Martha Kotey, Yakubu Alhassan, James Adomako, Godwin Nunoo-Mensah, Farzana Kapadia, Bismark Sarfo

**Affiliations:** 1 Department of Epidemiology and Disease Control, School of Public Health, University of Ghana, Legon, Accra, Ghana,; 2 Department of Health Policy, Planning and Management, School of Public Health, University of Legon, Accra, Ghana,; 3 Department of Plant and Environmental Biology, University of Ghana, Legon, Accra, Ghana,; 4 Pantang Hospital Antiretroviral Center, Pantang, Accra, Ghana,; 5 Department of Epidemiology, School of Global Public Health, New York University, New York, USA

**Keywords:** Comorbidities, antiretroviral therapy, Ghana

## Abstract

**Introduction:**

uptake of antiretroviral therapy (ART) and retention in care are associated with increased life expectancy but increased the risk of comorbid conditions in persons living with HIV (PLWH) and taking antiretroviral drugs. This study describes comorbid conditions among PLWH in Ghana.

**Methods:**

PLWH (n=222) out of a sample population of 900, randomly selected at Pantang ART Center participated in the study from June to July of 2020. Socio-demographic characteristics, HIV biomarkers, medication type and adherence, and diagnostic confirmed chronic conditions were extracted from medical records of PLWH. Cox proportional-hazard models and Kaplan-Meier curves graphing risk of experiencing comorbid conditions were performed. Log-rank test was performed at p<0.05.

**Results:**

fifty three point two percent of PLWH (222) experienced a comorbid condition including, respiratory conditions (17.6%), anaemia (12.2%), hypertension (12.2%), cardiovascular diseases (10.8%),and neurological conditions (10.8%).Factors associated with some of these conditions were medication adherence (aHR=0.43, 95% CI: 0.21-0.90) and visual changes (aHR=2.64, 95% CI: 1.08-6.45) for respiratory conditions, age (aHR=10.03, 95% CI; 1.22-82.37) for hypertension, and World Health Organization (WHO) clinical stages (stage II (aHR=13.36, 95% CI=1.54-115.63) and III (aHR=11.71, 95% CI=1.41-97.26))for peripheral neuropathy. Kaplan-Meier curves show significant risk of comorbid conditions for age, CD4 count ≤350 cells/mm^2^, WHO clinical stages III and IV, and ART non-adherence.

**Conclusion:**

understanding the types of comorbidities in PLWH is integral to providing feedback to primary care providers to monitor.

## Introduction

Based on the Joint United Nations Program on HIV/AIDS (UNAIDS) estimates, the number of people living with HIV (PLWH) in Ghana increased from 290,000 in 2010 to 340,000 in 2019 [1] This increase is partially attributed to Ghana's strides in decreasing gaps in the HIV care continuum for PLWH. Greater accessibility of and adherence to antiretroviral therapy (ART) have led to significant increases in life expectancy among PLWH [1-9]. In general, the increased life expectancy among PLWH in low- and middle-income countries (LMICs) has implication for increased morbidity and mortality associated with chronic comorbidities [2,10]. Specifically, PLWH on ART are at risk of experiencing cardiovascular disease, renal disease, liver disease, bone disease, and neurological conditions [11]. These chronic diseases, occur more in PLWH than in people who do not have HIV [12]. Among PLWH, these comorbid conditions may present at earlier ages. They will require additional care and monitoring to ensure that both the comorbid condition and HIV are managed appropriately and effectively, and this means care and monitoring over several decades.

The precise mechanism by which HIV is related to chronic diseases is not clear but studies have linked this to metabolic syndrome, pro-inflammatory and pro-thrombotic states [13]. Long-term inflammation because of persistent low-level viremia that may occur even with the use of ART have been associated with chronic diseases in PLWH [14]. Studies on chronic diseases and their risk factors among PLWH on ART have been predominantly conducted in high income countries. In fact, there is paucity of data on the presence of chronic comorbidities among PLWH in sub-Saharan Africa or the factors associated with their occurrence, despite the high burden of HIV and increasing access to ARTs in these regions. As life expectancy among PLWH increases due to greater access to ARTs, the presence of chronic conditions is likely to continue increasing [11]. A previous study has projected that by 2030, 28% of HIV-infected individuals would have more than three non-communicable diseases, and 54% will be on medications to treat these conditions [15]. To provide timely, context and country specific data and information to health care providers caring for PLWH, information on the presence of and risk factors for comorbid conditions is required to improve comprehensive care delivery as well as health and well-being for PLWH.

Previous studies have shown that there is elevation of inflammatory biomarkers among patients receiving care at the Pantang ART Center, which serves some of the sub-urban communities in the Greater Accra region of Ghana [16-17]. Inflammatory biomarkers are associated with many chronic disease conditions. Within the context of this study, we refer chronic or comorbid condition as disease condition (s) that is persistent over long period of time and require ongoing medical attention. These chronic conditions share common risk factors and they include hypertension, diabetes mellitus, cardiovascular diseases, chronic pulmonary diseases, neuropathological conditions and others such as oral and eye disorders and mental illness. Chronic conditions from genetic origins such as sickle cell and other haemoglobinopathies were excluded from this study. For the adult population in Ghana, a recent report indicated that the age-standardized prevalence of known cardiovascular risk factors was 15.1%, for diabetes mellitus, it was 26.1%, and 9.3% for hyperuricemia. Additionally, 10.1% of adults had peripheral artery disease, 8.3% had carotid thickening, 4.1% had left ventricular hypertrophy, and 2.5% had chronic kidney disease [18].

Meanwhile long-term exposures to antiretroviral medications have been implicated in renal impairment, increased metabolic dysfunction, lipodystrophy, and insulin resistance [19-22]. Indeed, various factors including socio-demographic, treatment, clinical and HIV-related could interact, and lead to the developing of chronic disease conditions in PLWH [23-25]. Against the background that there is increase in chronic disease condition in the general population, coupled with the paucity of data on chronic disease conditions in PLWH in Ghana, this study was warranted. Thus, this study examined the occurrence of chronic disease conditions and associated factors among PLWH receiving care at the Pantang ART Center.

## Methods

**Study design and sample:** for this retrospective cohort study, participants were identified from the patient population of the Pantang ART Center, located within Pantang Hospital. In 2008, the unit was designated as an ART Center and began offering full ART services. Currently the center provides HIV counseling and testing, antiretroviral therapy (ART), prevention of mother-to-child transmission (PMTCT) services and community health education about HIV prevention and treatment. There are over 900 HIV/AIDS patients including 20 children receiving ART services at the center.

To be eligible for this study, participants had to be ≥ 16 years old (16 years is considered as an adult age for HIV care in Ghana), linked to the Pantang ART center and receiving ART for at least three years and must be actively receiving care between January 2017 - December 2017. A three-year minimum for care was employed as it is likely that a chronic condition associated with HIV and or the ART use and adherence would manifest following this duration of ART use taking into consideration poor health seeking behavior and late diagnosis of HIV among the participants. Exclusion criteria were any prior diagnosis of tuberculosis, hepatitis B, other inflammatory conditions, diabetes, cardiovascular diseases, kidney disease or renal complications, or other chronic conditions prior to HIV seroconversion or ART uptake. These conditions were excluded so they do not confound any of the associations between the study variables in the current study. Medical records of HIV positive patients with missing observations due to either missed visits or missing data were also excluded.

The list of patients´ medical records (booklets) numbers of all the PLWH on ART was obtained from the ART center. A simple random process using the “sample” command in STATA version 16 was used to select record numbers from the list randomly. If numbers were selected and did not meet the inclusion and exclusion criteria, those particular records were replaced using another simple random selection procedure, excluding those already chosen records. This procedure was repeated until the required sample size was obtained and deemed eligible for further review.

The data were collected by Martha Kotey (MPH) who is a research assistant in the Department of Epidemiology and Disease Control of the School of Public Health, University of Ghana, and Godwin Nunoo-Mensah (RN, MPhil) who is an HIV counselor and Registered Nurse at the ART center of Pantang Hospital. They are all part of the study team and co-authors of the article.

**Sample size estimation:** prevalence of hypertension which is a commonly reported chronic/comorbid disease in both the general and PLWH was used to estimate the sample size for the study. A cohort study in Uganda estimated that the prevalence of hypertension in PLWH is 14.5% [26] (Mayanja *et al*., 2017). Using this expected prevalence and a 10% adjustment based on the assumption that some of the records from the health facility could have missing observations, the final sample size for the study was estimated to be 210.

**Medical record extraction:** the following information were extracted from the medical records of participants: socio-demographic characteristics including sex, age, occupational status, educational level, religious affiliation and marital status; behavioral factors such as sexual activity, regular condom use, and cigarette smoking and physical and clinical characteristics including weight (kg), first clinic visit, HIV type, tuberculosis status, drug allergies, WHO HIV clinical stage, and CD4+ cell count.

For ART use, we extracted information on prescription of the following drug regimens: Tenofovir+Lamivudine+Nevirapine(TDF+3TC+NVP); Zidovudine+Lamivudine+Efavirenz (AZT+3TC+EFV); Tenofovir (TDF) +Emtricitabine (FTC) + Nevirapine (NVP); Zidovudine + Lamivudine + Nevirapine (AZT+3TC+NVP) and Tenofovir + Lamivudine + Efavirenz (TDF+3TC+EFV). In addition to use, we also extracted and recorded changes in HIV treatment regimens. Adherence to ART regimens was determined based on participant report of missing medication doses in the 3 days preceding hospital visit based on the Ghana Health Service clinical guidelines for adherence to HIV medication which is clearly outlined in the patient´s medical folder (record) [27]. And this was recorded as 0 missed doses, 1-2 missed doses, 3-4 missed doses, and >5 missed doses. Data on adherence was recorded for visits during the previous three years. Participants who reported missing 0 doses during each visit over the prior three-year period were categorized as adherent while those who reported missing 1->5 doses across visits were categorized as non-adherent. Adherence to comorbid disease medications were not reported in participants´ medical records so these could not be determined.

Finally, information on the presence of HIV-related opportunistic infections and comorbidities was extracted for the preceding three-years. Chronic conditions investigated here include hypertension, peripheral neuropathy, diabetes, liver disease, kidney disease, cardiovascular disease, and respiratory tract infections. These disease conditions were recorded in the medical records of PLWH through laboratory and other diagnostic procedures and confirmed by the Physicians at the center. For instance, those with systolic and diastolic blood pressure of above 140mmHg and 90mmHg are being treated as hypertensive patients [28] and are put on various hypertensive medications. With diabetes for example, various screening and diagnostic criteria were recorded in patients´ medical records with values indicative of diabetes diagnosis where glycated hemoglobin (HbA1c) ≥ 6.5% (48 mmol/mol), fasting plasma glucose ≥ 126 mg/dL (7.0 mmol/L), and random plasma glucose ≥ 200mg/dL (11.1 mmol/L) were reported.

The participants with confirmed chronic conditions through laboratory and other diagnostic procedures were all undergoing treatment in addition to their antiretroviral therapy as prescribed by the clinicians at the ART center. A condition was considered newly diagnosed if at the visit prior to diagnosis, the participant was disease free for that condition. In addition to abstracting data on the onset of a comorbid condition, we also extracted the date of first diagnosis. To ensure data quality for the study, the extraction tool was pre-tested at a different ART center prior to the commencement of the study. Additionally, data were entered in excel and verified by two independent investigators of the study team to ensure data completeness and consistency.

**Data analysis:** first, exploratory data analyses were conducted to describe sociodemographic characteristics, behaviors, clinical characteristics, and ART regimens at the start of the record review period. We then examined distribution and onset of the comorbid conditions observed during the first three years of receiving care and these were also described using bar charts. Cox-proportional hazard model was employed to examine the risk of experiencing at least one chronic condition among the PLWH receiving care for at least three years. It was assumed that the effect of the hazard ratio for the groups has been constant overtime and this was tested through the goodness-of-fit test before the Cox regression analysis was performed. Factors that were statistically significantly related to the main outcome were included in multivariable Cox proportional hazard models, controlling for potential confounders, while assessing the effect of each confounding variable on the hazard of developing a chronic condition. The final model was determined using the backward stepwise elimination method. We started with the model that contains all the variables under consideration and the least significant variables were removed one at a time. Only the statistically significant variables (p<0.05) were left in the final model. The Kaplan-Meier curves graphing risk of experiencing at least one chronic disease condition were plotted to identify differences in risk of developing a comorbid condition by key sociodemographic and clinical characteristics. The log-rank test was used to test the equality of hazard curves with p-value < 0.05 as significance. All analyses were conducted using STATA 16 (College Station, TX).

**Ethical approval:** this study received ethical clearance from the Ghana Health Service Ethics Review Committee (GHS-ERC 017/02/20) and permission was granted by the Pantang ART Center to conduct medical record abstraction. Participants´ privacy and confidentiality were protected by using coded numbers in identifying their medical records. None of the participants were contacted for any information in this study. Soft copies of the data were password protected on the laptop and hard copies were stored in cabinets with access granted to only the research team.

**Consent for publication:** consent and permission were obtained for the publication of these data from the participants and the Pantang ART clinic.

**Availability of data and materials:** the datasets used and/or analysed during the current study are available from the corresponding author on reasonable request.

## Results

Among the 222 participants who participated in this study, the average age was 39 years (SD=9.64 years), 65.3% were female, 42.8% were married, 82.8% were employed, and 47.8% had a junior secondary school level of education. Approximately, 18.5% of participants were recorded as having a weight of <50 kgs. While most patients were diagnosed with HIV-1 (72.9%), for 23.9% of the sample, HIV type was unknown. Additionally, 32.9% of patients were reported as having Stage III/IV HIV and 60.4% had a CD4 cell count below 350 cells/mm^3^ (mean±SD = 244.6±19.7).

About two-thirds of participants were on a 1^st^ line/1^st^ choice ART regimen with 40.1% receiving Tenofovir+Lamivudine+Efavirenz (TDF+3TC+EFV). Slightly more than half of the sample (52.7%) reported adequate ART adherence, with 31.9% identified as having had a change to their ART regimen during the first three years in care. The most common physical health concerns reported at the visit prior to ART uptake included severe weight loss, chronic cough, chills, skin rash and persistent headache. Finally, in terms of behavioral factors, 7.2% of patients reported ever smoked cigarette, 45.5% reported being sexually active and 79.7% reported inconsistent condom use (Table 1).

**Table 1:** distribution of baseline socio-demographic, physical, clinical and behavioral characteristics of study participants, n=222, Accra, Ghana

	(n)	%
**Age** (mean ± SD)		39.0 ± 9.6
**Sex** (% female)	145	65.3
**Marital status**		
Never married	59	26.6
Cohabiting	18	8.1
Married	95	42.8
Divorced/separated/widowed	50	22.5
**Employed (yes)**	184	82.9
**Education**		
None	34	15.3
Primary	28	12.6
JSS/MSLC	106	47.8
Secondary/technical/vocational	36	16.2
Tertiary	18	8.1
**Weight, kg (mean ± SD)**		(58.2 ± 11.2)
<50	41	18.5
50-70	149	67.1
>70	23	10.4
**HIV type**		
HIV I	162	72.9
HIV I and II	7	3.2
Unknown	53	23.9
**WHO clinical stage**		
Stage I	47	21.2
Stage II	29	13.1
Stage III	44	19.8
Stage IV	29	13.1
Unknown	73	32.9
**CD4 cell count level, cells/mm^3^**		
<350 cells/mm^3^	134	60.4
350 cells/mm^3^	42	18.9
Unknown	46	20.7
**Prescribed drug regimen**		
1^st^ line/1^st^ choice ART regimen	143	64.4
2^nd^ line/2^nd^ choic ART regimen	79	35.6
**Drug combinations**		
TDF+3TC+EFV	89	40.1
TDF+ FTC+NVP	17	7.7
TDF+3TC+NVP	15	6.8
AZT+3TC+EFV	17	7.7
AZT+3TC+NVP	45	20.3
Other combination	39	17.6
**Adherence to ARV medication**		
Non-adherent	105	47.3
Adherent	117	52.7
**Change in ART medication**		
Yes	71	31.9
No	151	68.0
**Smoking status**		
Never smoked	206	92.8
Ever smoked	16	7.2
**Sexually active**		
Yes	101	45.5
No	117	52.7
**Regular condom use**		
Yes	24	10.8
No	177	79.7

**Figure 1:**
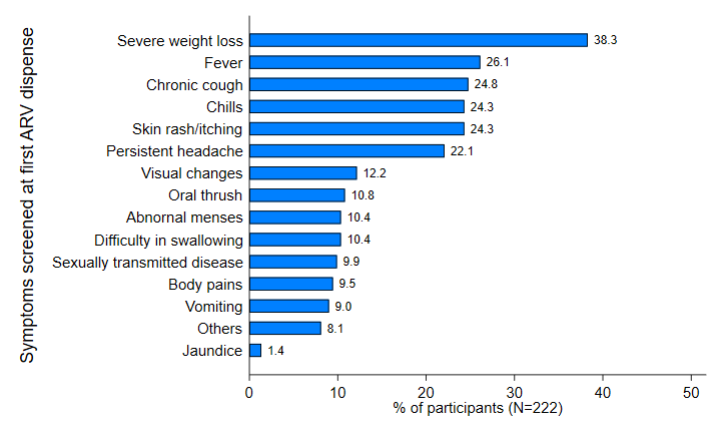
conditions screened before ART uptake by study participants

Figure 1 shows the clinical conditions screened before ART uptake, demonstrating that the conditions with the highest percentages were severe weight loss (38.83%), fever (26.1%), chronic cough (24.8%), chills (24.3%), and skin rash itching (24.3%). The conditions with the least percentages were other STI (9.9%), body pains (9.5%), vomiting (9%), other conditions (8.1%) and jaundice (1.4%). None of the conditions screened before ART uptake had any statistically significant association with the experience of chronic conditions in our cox-proportional regression model (data not shown).

**Figure 2:**
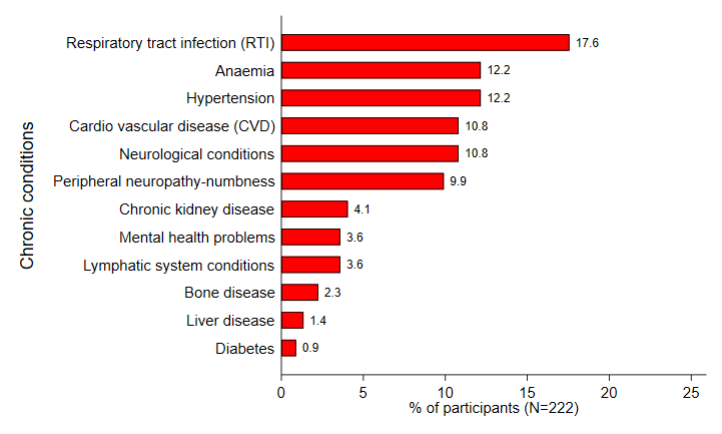
chronic conditions developed three years or more of receiving care and ART uptake

During the first three years in care and ART uptake, 17.6% of the patients developed respiratory tract infections, 12.2% developed anemia and hypertension, 10.8% developed cardiovascular diseases and neurological conditions, and 9.9% developed peripheral neuropathy. 4.1% developed chronic kidney conditions, and less common chronic conditions identified included lymphatic system conditions (3.6%), bone disease (2.3%), liver diseases (1.4%), and diabetes (0.9%). Overall, 53.2% of patients were identified as having developed at least one chronic condition in the 3 years post care and ART uptake (Figure 2).

The overall age of patients was significantly associated with the risk of developing chronic condition(s) in the first three years of receiving care and ART uptake in both the unadjusted (p=0.013) and adjusted model (p=0.015). The adjusted cox-proportional regression model shows that patients aged 40-49 had two times increased hazard of having a chronic condition (aHR=2.17, 95% CI: 1.04-4.54, p<0.05) compared to those aged below 30 years while patients older than 49 years had over three times increased hazard of developing chronic condition(s) (aHR=3.34, 95% CI=1.43-7.76, p<0.01). The adjusted cox-proportional regression models for the other variables were not significant (Table 2).

**Table 2:** socio-demographic factors associated with chronic conditions in the sample of PLWH

		Chronic conditions	Unadjusted cox-proportional model		Adjusted cox-proportional model	
Variables	N	n (%)	uHR [95% CI]	P-value	aHR [95% CI]	P-value
**Total**	**222**	119 (53.6)				
**Age**				0.013*		0.015*
<30	35	13 (37.1)	1.00 [reference]		1.00 [reference]	
30-39	76	37 (48.7)	1.40 [0.75-2.64]		1.28 [0.64-2.55]	
40-49	79	46 (58.2)	2.10 [1.14-3.90]*		2.17 [1.04-4.54]*	
>49	32	23 (71.9)	2.58 [1.31-5.11]**		3.34 [1.43-7.76]**	
**Sex**				0.314		
Male	77	36 (46.8)	1.00 [reference]		-	
Female	145	83 (57.2)	1.22 [0.83-1.81]		-	
**Marital status**				0.044*		0.236
Married	95	45 (47.4)	1.00 [reference]		1.00 [reference]	
Never married	59	31 (52.5)	1.09 [0.69-1.73]		1.17 [0.68-2.02]	
Cohabiting	18	10 (55.6)	1.45 [0.73-2.88]		2.14 [1.01-4.52]*	
Divorced/separated	50	33 (66.0)	1.60 [1.02-2.51]*		1.26 [0.75-2.10]	
**Occupation**				0.065		
Employed	184	94 (51.1)	1.00 [reference]		-	
Unemployed	38	25 (65.8)	1.52 [0.97-2.36]		-	
**Education**				0.012*		0.258
None	34	22 (64.7)	3.45 [1.31-9.13]*		3.28 [1.17-9.21]*	
Primary	28	16 (57.1)	2.57 [0.94-7.03]		2.66 [0.91-7.74]	
JSS/MSLC	106	58 (54.7)	2.70 [1.08-6.74]*		2.51 [0.98-6.44]	
SHS/TECH/VOC.	36	18 (50.0)	2.39 [0.89-6.44]		2.79 [1.00-7.77]	
Tertiary	18	5 (27.8)	1.00 [reference]		1.00 [reference]	
**Dependent children**				0.471	-	
None	106	59 (55.7)	1.00 [reference]		-	
One	55	30 (54.5)	1.06 [0.68-1.65]		-	
Two	34	13 (38.2)	0.71 [0.39-1.29]		-	
>Two	27	17 (63.0)	1.26 [0.73-2.15]		-	
**Weight in kg**				0.225	-	
<50	41	21 (51.2)	1.43 [0.65-3.11]		-	
50-70	149	85 (57.0)	1.76 [0.88-3.49]		-	
>70	23	9 (39.1)	1.00 [reference]		-	
**Smoking status**				0.847	-	
Never smoked	206	111 (53.9)	1.00 [reference]		-	
Ever smoked	16	8 (50.0)	0.93 [0.45-1.91]		-	

uHR: unadjusted harzard ratio. aHR: adjusted hazard ratio. CI: confidence interval. P-value notation **(based on null hypothesis of equal proportion**): *: p<0.05. **: p<0.01. ***: p<0.001

Medication adherence and visual changes are the factors that showed significant association with the experience of respiratory tract infection (RTI) during the first three years of care. The respective adjusted model are 57% reduced risk of RTI for PLWH who were medication adherent (aHR=0.43, 95% CI: 0.21-0.90, p=0.025), and over two times increased with visual changes (aHR=2.64, 95% CI: 1.08-6.45, p=0.033) (Table 3).

**Table 3:** factors associated with respiratory tract infection in the first three years on ARV

	Respiratory tract infections					
	RTI		Unadjusted cox-proportional model		Adjusted cox-proportional model	
Variables	N	n (%)	aHR [95% CI]	P-value	aHR [95% CI]	P-value
Total	222	39 (17.57)				
**CD4 below 350**				0.078		0.383
Yes	134	30 (22.39)	1.00 [reference]		1.00 [reference]	
No	42	6 (14.29)	0.60 [0.25-1.45]		0.99 [0.38-2.56]	
Unknown	46	3 (6.52)	0.28 [0.09-0.93]*		0.41 [0.11-1.47]	
**Drug combinations**				0.152		0.155
Other combination	39	3 (7.69)	1.00 [reference]		1.00 [reference]	
TDF+3TC+EFV	89	14 (15.73)	2.32 [0.67-8.07]		3.51 [0.96-12.79]	
TDF+ FTC+NVP	17	6 (35.29)	6.16 [1.54-24.67]*		6.66 [1.51-29.25]*	
TDF+3TC+NVP	15	4 (26.67)	3.72 [0.83-16.62]		4.58 [0.96-21.93]	
AZT+3TC+EFV	17	4 (23.53)	3.64 [0.82-16.28]		4.55 [0.97-21.44]	
AZT+3TC+NVP	45	8 (17.78)	2.55 [0.68-9.60]		2.22 [0.58-8.57]	
**Adherence to ARV medication**				0.005		0.025
Non-adherent	105	27 (25.71)	1.00 [reference]		1.00 [reference]	
Adherent	117	12 (10.26)	0.38 [0.19-0.74]**		0.43 [0.21-0.90]*	
Fever				0.006		0.351
No	164	22 (13.41)	1.00 [reference]		1.00 [reference]	
Yes	58	17 (29.31)	2.43 [1.29-4.58]**		1.45 [0.66-3.19]	
**Severe weight loss**				0.025		0.810
No	137	18 (13.14)	1.00 [reference]		1.00 [reference]	
Yes	85	21 (24.71)	2.05 [1.09-3.85]*		1.10 [0.50-2.44]	
**Sexually transmitted infections**				0.048		0.672
No	200	32 (16.00)	1.00 [reference]		1.00 [reference]	
Yes	22	7 (31.82)	2.29 [1.01-5.18]*		1.23 [0.48-3.17]	
**Visual changes**				0.025		0.033
No	195	30 (15.38)	1.00 [reference]		1.00 [reference]	
Yes	27	9 (33.33)	2.35 [1.11-4.95]*		2.64 [1.08-6.45]*	

uHRR: unadjusted hazard ratio. aHR: adjusted hazard ratio. CI: confidence interval. P-value notation (**based on null hypothesis of equal proportion**): *: p<0.05. **: p<0.01. ***: p<0.001

**Figure 3:**
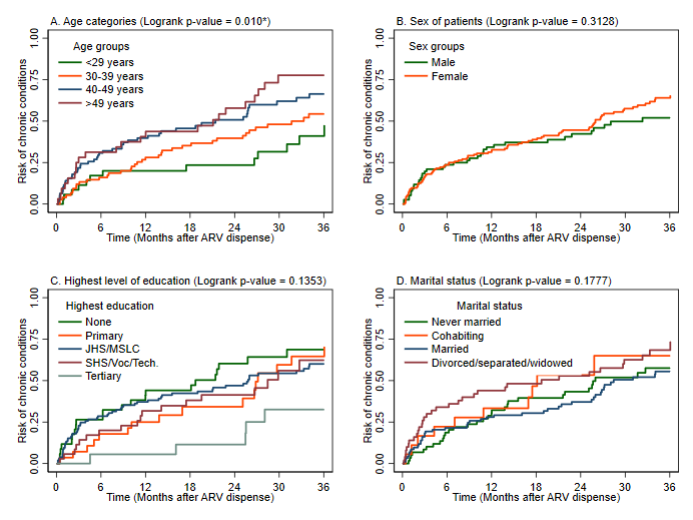
Kaplan Meier curves of the hazard of developing chronic condition(s) in PLWH for selected socio-demographic factors (age categories (A); sex of patients (B); highest level of education (C); marital status (D))

Table 4 shows that age is the only variable that was significantly associated with hypertension among the PLWH. The cox regression shows significant associations for both the unadjusted (uHR)=9.37, (95% CI; 1.17-74.99, p=0.022), and adjusted (aHR)=10.03, (95% CI; 1.22-82.37, p=0.026) model between age and hypertension. The small sample size within the sub-group accounted for the wide confidence interval. Table 5 shows the result for our cox-proportional models demonstrating significant association between PLWH diagnosed with WHO stages II (aHR=13.36, 95% CI=1.54-115.63, p<0.05) and III (aHR=11.71, 95% CI=1.41-97.26, p<0.05), and peripheral neuropathy. The wide confidence intervals are attributed to the small sample size within the sub-groups. Kaplan Meier (Figure 3) curves show an increased hazard of chronic conditions in participants for older age groups with statistically significant differences in risk of complications (p=0.010). The curves were not statistically significantly different across sex, highest level of education, and patient marital status (p>0.05).

**Table 4:** factors associated with hypertension in the sample of PLWH in the first three years of care

		Hypertension				
	Hypertension		Unadjusted cox-proportional model		Adjusted cox-proportional model	
Variables	N	n (%)	uHR [95% CI]	P-value	aHR [95% CI]	P-value
Total	222	27 (12.16)				
**Age group**				0.022*		0.026*
<29	35	1 (2.86)	1.00 [reference]		1.00 [reference]	
30-39	76	5 (6.58)	2.20 [0.26-18.81]		2.40 [0.27-20.99]	
40-49	79	13 (16.46)	6.36 [0.83-48.61]		7.10 [0.92-55.01]	
>49	32	8 (25.00)	9.37 [1.17-74.99]*		10.03 [1.22-82.37]*	
**Drug combinations**				0.799		0.661
Other combination	39	4 (10.26)	1.00 [reference]		1.00 [reference]	
TDF+3TC+EFV	89	11 (12.36)	1.43 [0.45-4.49]		1.68 [0.53-5.37]	
TDF+ FTC+NVP	17	1 (5.88)	0.55 [0.06-4.96]		0.72 [0.08-6.63]	
**TDF+3TC+NVP**	15	3 (20.00)	2.12 [0.47-9.47]		3.39 [0.74-15.62]	
AZT+3TC+EFV	17	3 (17.65)	1.77 [0.40-7.92]		1.62 [0.36-7.34]	
AZT+3TC+NVP	45	5 (11.11)	1.03 [0.28-3.85]		1.40 [0.36-5.37]	
**Adherence to ARV medication**				0.299		0.552
Non-adherent	105	11 (10.48)	1.00 [reference]		1.00 [reference]	
Adherent	117	16 (13.68)	1.50 [0.70-3.24]		1.27 [0.57-2.83]	

uHR: unadjusted hazard ratio. aHR: adjusted hazard ratio. CI: confidence interval. P-value notation (**based on null hypothesis of equal proportion**): *: p<0.05. **: p<0.01. ***: p<0.001

**Table 5:** factors associated with peripheral neuropathy in the sample of PLWH in the first three years of care

		Peripheral Neuropathy (PN)				
	PN		Unadjusted cox-proportional model		Adjusted cox-proportional model	
Variables	N	n (%)	uHR [95% CI]	P-value	aHR [95% CI]	P-value
Total	222	22 (9.91)				
**WHO clinical stage**				0.07		0.05
Stage I	47	1 (2.13)	1.00 [reference]		1.00 [reference]	
Stage II	29	6 (20.69)	10.63 [1.28-88.35]*		13.36[1.54-115.63]*	
Stage III	44	7 (15.91)	8.33 [1.02-67.71]*		11.71[1.41-97.26]*	
Stage IV	29	4 (13.79)	6.32 [0.71-56.58]		6.89 [0.76-62.70]	
Unknown	73	4 (5.48)	2.67 [0.30-23.91]		3.43 [0.38-31.08]	
**Drug combinations**				0.44		0.45
Other combination	39	3 (7.69)	1.00 [reference]		1.00 [reference]	
TDF+3TC+EFV	89	7 (7.87)	1.15 [0.30-4.46]		1.41 [0.35-5.69]	
TDF+ FTC+NVP	17	4 (23.53)	3.35 [0.75-14.99]		3.40 [0.72-16.01]	
**TDF+3TC+NVP**	15	2 (13.33)	1.80 [0.30-10.77]		3.23 [0.50-20.76]	
AZT+3TC+EFV	17	0 (0.00)	(empty)	.	(empty)	.
AZT+3TC+NVP	45	6 (13.33)	1.81 [0.45-7.24]		2.18 [0.53-8.89]	
**Adherence to ARV medication**				0.06		0.10
Non-adherent	105	15 (14.29)	1.00 [reference]		1.00 [reference]	
Adherent	117	7 (5.98)	0.42 [0.17-1.03]		0.46 [0.18-1.15]	

uHR: unadjusted hazard ratio. aHR: adjusted hazard ratio. CI: confidence interval. P-value notation (**based on null hypothesis of equal proportion):** *: p<0.05. **: p<0.01. ***: p<0.001

**Figure 4:**
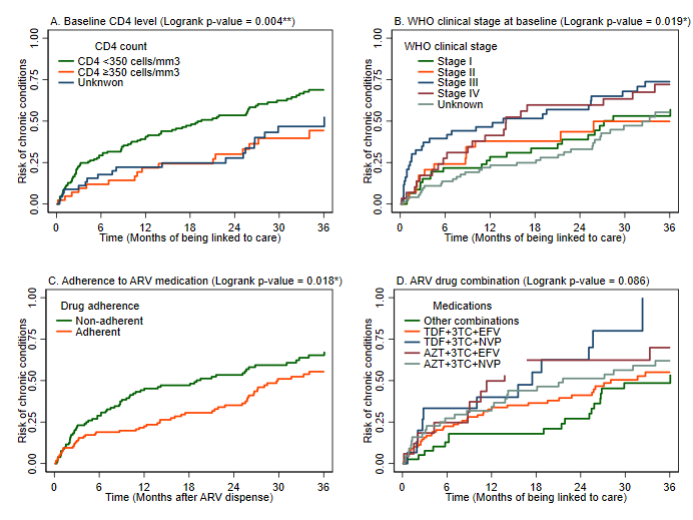
Kaplan Meier curves of the hazard of developing chronic condition(s) in PLWH for selected clinical factors (baseline CD4 level (A); WHO clinical stage at baseline (B); adherence to ARV medication (C); ARV drug combination (D))

Figure 4 shows Kaplan-Meier curves (under baseline CD4 levels) demonstrating a higher hazard of developing a chronic condition among PLWH with a CD4 count ≤350 cells/mm^3^ compared to those with CD4 >350 cells/mm^3^ or those with unknown CD4 values at baseline (p=0.004). Additionally, the hazard curves under WHO clinical stage at baseline show increased hazard of developing chronic condition(s) among those at WHO clinical stages III and IV compared to those at WHO clinical stages I and II or unknown stage (p=0.019). While there was no statistically significant difference in the hazard functions by ART drug combination, the hazard curves were significantly different for ART adherence. Under adherence to antiretroviral (ARV) medication, the curves show an increased in developing comorbid condition by PLWH who were non-adherent to medication compared to those who adhere to their medication regimen (p=0.018).

## Discussion

This study assessed the prevalence of comorbidities and their associated factors in a sample of PLWH receiving care for at least three years at the Pantang ART Center. Our findings show that more than half of the participants developed at least one chronic disease condition within the first three years of care and ART uptake. Some of the chronic conditions developed were respiratory tract infection (RTI), anemia, hypertension, cardiovascular disease (CVD), and peripheral neuropathy. Regarding the clinical characteristics of participants, CD4 count, WHO clinical stage, and adherence to medication were associated with developing chronic conditions. With sociodemographic factors, only age of the participants was significantly associated with these comorbid conditions. The respective factors associated with each of the condition under sub-group analysis were medication adherence and visual changes for respiratory tract infection, age for hypertension, and WHO clinical stages for peripheral neuropathy.

A study conducted in Brazil has shown high incidence of respiratory tract infection in PLWH receiving ART [29]. Important predictors of respiratory tract infection in this Brazilian study were found to be viral load and CD4 counts. Although viral load was not assessed in our study, the CD4 counts of majority of the study participants were found to be lower than 350cells/mm^3^. With such low CD4 counts, the ability of the immune system to work against opportunistic infections such as respiratory tract infection is compromised, and this could be attributed to the observed findings in this study. Furthermore, medication adherence and visual changes were the factors associated with respiratory tract infection in the current study.

Findings from the Strategies for Management of Antiretroviral Therapy (SMART) study indicated that PLWH who were non-adherent to medication had a significantly greater risk of developing chronic diseases than those who were adherent [30]. The use of antiretroviral medications suppresses viral load in PLWH and for this to be achieved, patients need about 95% adherence level [30]. Meanwhile, symptoms of respiratory tract infection such as sneezing, coughing, and fever can have some association with visual changes through the transmission of pathogens via nasal passages to the eyes. With hypertension, studies conducted in Tanzania and other parts of Africa have demonstrated the development of hypertension in PLWH who are on ART [31-33]. Adults PLWH on ART have been reported to have a higher incidence of hypertension [31-33] which is consistent with the findings from this study, where our sub-group analysis shows that age was significantly associated with hypertension among the PLWH. Studies around the world have demonstrated that 35% of all HIV-infected adults on ART have hypertension and over 50% of those on ART who are older than 50 years, have hypertension [34].

The findings from this study implicating CVD in PLWH, is in line with another study which has reported that the use of anti-HIV drugs is associated with CVD risk [35]. Findings from the Fat Distribution in Women with HIV Infection (FRAM) cohort study also indicated that HIV infection is an independent risk factor for atherosclerosis, similar in magnitude to traditional CVD risk factors, such as smoking and advancing age [36]. As PLWH experience a decrease in AIDS-related mortality, CVDs have become the most common cause of death after cancer with those whose viral load is undetected [37]. The other chronic disease condition that was identified among the participants in this study is peripheral neuropathy. About 9% of the participants have developed peripheral neuropathy while on HIV medication in this study, although this appears to be lower compared with what has been previously reported in other studies in Cameroon [38] and South Africa [39]. Peripheral neuropathy is one of the common complications associated with HIV infection. The proposed mechanism for this complication stemmed from immunopathogenesis associated with neurotoxicity from the HIV virus and its products, as well as the neurotoxicity from the adverse effects of the HIV medications. In the current study, for those who developed peripheral neuropathy during the first three years in care, there was an increased risk for patients who were at WHO clinical stages II and III. One of the most frequent neurological complications among PLWH is distal symmetry polyneuropathy, which affects about 50% of all HIV infected population [40]. Low CD4+ T-cells <500cells/mm^3^ is associated with an increased risk of developing peripheral neuropathy [41] and the CD4+ T cells of majority of the participants in this study is low.

Compared to those with WHO HIV clinical stage I, the risk of developing a chronic condition was higher among those with stage III. At HIV stage III, the person´s immune system is severely impaired [42]. Furthermore, due to lack of uptake of HIV testing in the general population, most people do not test early to know their HIV status until they fall sick, by which time they might have been getting to the later stages of the infection, where adverse symptoms have already started. At this stage, the immune system is weakened, and as such, the individual becomes more prone to other chronic conditions. This is consistent with other findings which indicated that stage III is characterized by severe weight loss, chronic diarrhea, fever, oral candidiasis, and CD4+ cell count <200 cells/mm^3^ [43]. In this sample of PLWH, age was found to be a factor which is consistently associated with comorbid condition(s) in the participants. As people advance in age, they become prone to developing an age-related chronic condition which is exacerbated by HIV infection and the adverse effect of antiretroviral medications. With increasing ART coverage, more people will have access to treatment options and as the years of survival of PLWH increases, more patients will move to age groups with higher incidence of poor health outcomes. Due to this paradigm shift, PLWH are faced with a greater risk of developing chronic conditions and as such HIV service providers should work collaboratively with specialists to help manage these chronic disease conditions in their patients. The study has demonstrated high prevalence of chronic conditions in PLWH receiving care, and as such there is the need for routine screening for not only opportunistic infections but also for chronic non-communicable diseases even as their CD4 counts increase and their viral loads remained undetectable.

**The findings from this study should be interpreted within the context of the following limitations:** the follow-up interval was not consistent for all patients. Some patients did not regularly visit the hospital; therefore, dates of visits were not compatible with their scheduled visit date in their medical records. This could interfere with the date that a chronic disease was recorded. The period spent on ART was an estimate of at least 3 years. The exact fraction of time spent on ART was not calculated and the study could not control for the duration an individual had lived with HIV during the study period. And we also admit the limitation of self-reported adherence to medication. These factors potentially determine the acquisition of chronic illness among PLWH. Additionally, we could not explicitly determine the timing of diagnosis of HIV in relation to the chronic illness in the participants. Despite these limitations, our study provides important findings related to chronic diseases in PLWH. Our findings highlight the importance of continuous screening for chronic diseases in PLWH even with high CD4 counts and undetectable viral load.

## Conclusion

In this study more than half of the PLWH had chronic disease conditions at least during the first three years of being linked to HIV care. The observed chronic disease conditions were respiratory tract infection (RTI), anemia, hypertension, cardiovascular disease (CVD), and peripheral neuropathy. The risk of developing these conditions was higher for older age group, CD4 counts ≤350 cells/mm^3^, WHO clinical stages III and IV, and non-adherent to ART medication. PLWH on ART at the Pantang ART and similar facilities must be monitored closely by clinicians for increased risk of chronic or comorbid conditions. At the Pantang ART center, both PLWH and patients seeking general medical treatment receive care from the same primary health physician. This integrated primary health and HIV care model is being piloted by Ghana Health Service to reduce the stigma associated with HIV treatment. Thus, given that this model is in place, understanding the development of chronic comorbidities is integral to providing adequate feedback and guidance to primary care providers on the types of chronic conditions to monitor to ensure coordination around medications for these conditions and ARTs to prevent side-effects and medication burden and to make sure that adherence to all medications is optimized. The reports from this study have important implication for improved quality of life for PLWH in Ghana and sub-Saharan Africa and the following recommendations are provided: clinicians at the Pantang ART and similar centers must closely monitor PLWH for the following chronic conditions: respiratory tract infection (RTI), anemia, hypertension, cardiovascular disease (CVD), and peripheral neuropathy when they are linked to care and ART medication adherent must be closely monitored by HIV counselors and clinicians, since non-adherent to medication predisposes PLWH to developing comorbid conditions.

### What is known about this topic

- The development of chronic disease conditions among people living with HIV;
- Hypertension especially has been identified to be associated with PLWH.

### What this study adds

- The variety of chronic conditions that are developed after patients are linked to HIV care;
- The various demographic factors that are associated with the development of these chronic conditions;
- The antiretroviral therapy and adherence to medications being associated with these chronic conditions.
